# Associations between Depressive State and Impaired Higher-Level Functional Capacity in the Elderly with Long-Term Care Requirements

**DOI:** 10.1371/journal.pone.0127410

**Published:** 2015-06-02

**Authors:** Soshiro Ogata, Chisato Hayashi, Keiko Sugiura, Kazuo Hayakawa

**Affiliations:** 1 Support Office for Female Researchers, Osaka City University, Osaka, Japan; 2 Department of Public Health Nursing, Graduated School of Nursing, Osaka City University, Osaka, Japan; 3 Faculty of Nursing, Senri Kinran University, Osaka, Japan; 4 Mie Prefectural College of Nursing, Mie, Japan; Federal University of Rio de Janeiro, BRAZIL

## Abstract

Depressive state has been reported to be significantly associated with higher-level functional capacity among community-dwelling elderly. However, few studies have investigated the associations among people with long-term care requirements. We aimed to investigate the associations between depressive state and higher-level functional capacity and obtain marginal odds ratios using propensity score analyses in people with long-term care requirements. We conducted a cross-sectional study based on participants aged ≥65 years (n = 545) who were community dwelling and used outpatient care services for long-term preventive care. We measured higher-level functional capacity, depressive state, and possible confounders. Then, we estimated the marginal odds ratios (i.e., the change in odds of impaired higher-level functional capacity if all versus no participants were exposed to depressive state) by logistic models using generalized linear models with the inverse probability of treatment weighting (IPTW) for propensity score and design-based standard errors. Depressive state was used as the exposure variable and higher-level functional capacity as the outcome variable. The all absolute standardized differences after the IPTW using the propensity scores were <10% which indicated negligible differences in the mean or prevalence of the covariates between non-depressive state and depressive state. The marginal odds ratios were estimated by the logistic models with IPTW using the propensity scores. The marginal odds ratios were 2.17 (95%CI: 1.13–4.19) for men and 2.57 (95%CI: 1.26–5.26) for women. Prevention of depressive state may contribute to not only depressive state but also higher-level functional capacity.

## Introduction

For the elderly, higher-level functional capacity is an essential competence to sustain healthy and socially independent living [1–4]. It includes preparing meals, handling money, writing forms, using public transport, reading books or newspapers, and visiting friends’ homes [1,2,4]. Declines in higher-level functional capacity have been reported to be associated with cognitive impairment [5] and mortality [1], for example, people with impaired higher-level functional capacity have been reported to have higher one-year mortality rate (10%–20%) than people without impaired higher-level functional capacity (<5%) [1].

Many previous studies have shown that depressive state was associated with functional capacity measured by basic activities of daily living (BADL) [6–9], such as dressing, ambulation, going to the toilet, or eating, and that measured by instrumental ADL (IADL) [7,8,10–12], such as shopping, food preparation, or housekeeping. Although higher-level functional capacity generally deteriorates before the onset of declines in BADL [1], there have been not many studies investigating the associations between depressive state and higher-level functional capacity [13–15]. Although the previous studies have shown the significant associations among community-dwelling elderly, few studies have examined the associations among those requiring long-term care. Among those requiring long-term care, preventing impaired higher-level functional capacity is important to maintain independent daily living. In fact, to maintain independent daily living among people requiring long-term, the Japanese government has implemented the Long-Term Care Insurance Act beacuse in the near furture, an unprecedented super-aged population is expected in Japan (Act No. 123 of December 17, 1997).

In addition, the previous studies investigating the associations between depressive state and higher-level functional capacity have estimated conditional effects, which were the average effects of an exposure on an outcome at an individual-level and are usually estimated by traditional regression models [16,17]; to date, none has estimated marginal effects, which were the average effects of an exposure on an outcome at a population level and are usually estimated by randomized controlled trials (RCTs) and propensity score analyses [16,17]. Not only the conditional effects but also the marginal effects of any depressive state on functional capacity are important considerations, and especially, the marginal effects are useful to apply results of researches to clinical settings at population level.

We hypothesized that depressive state could cause impaired higher-level functional capacity. Therefore, we investigated the association between depressive status and higher-level functional capacity and obtain marginal odds ratios using propensity score analyses in people requiring long-term care.

## Materials and Methods

### Participants

We conducted a cross-sectional study and recruited participants who used outpatient care services for long-term preventive care via the Arataka Outpatient Services in Hyogo, Japan. Outpatient services for long-term preventive care, a component service of the Long-Term Care Insurance Act, are often used by people requiring long-term care. The eligible criteria for the present study were men and women aged ≥65 years, without dementia, and not currently receiving any treatment for depression. Unsigned self-report questionnaires were distributed to the participants in November 2012.

### Ethics statement

Participants provide verbal informed consent to participate in the present study. We explained to participants that participants were considered to provide agreement to participate in the present study if they voluntarily answered and handed in the unsigned self-report questionnaires. The consent procedure and the present study were approved by the Ethics Committee of the Faculty of Nursing at Senri Kinran University.

### Measurements

#### Higher-level functional capacity

The Tokyo Metropolitan Institute of Gerontology (TMIG) index of competence [1] was used to measure higher-level functional capacity. Participants answered each question with “yes” or “no.” The total score of the TMIG-index (range 0–13; worst to best) was indicated by the number of items answered “yes.” The total TMIG-index score has been reported to be inversely associated with 1-year mortality [1], people with the total score ≤5 having a 10%–20% 1-year mortality, whereas those with the total score >5 having a <5% 1-year mortality [1]. Participants with the total score of TMIG-index ≤5 were considered to have impaired higher-level functional capacity and increased 1-year mortality. Higher-level functional capacity was assessed as a binary variable based on this cut-off point [1].

#### Depression status

To assess depressive state, the 15-item version of the Geriatric Depression Scale (GDS) was used [18]. Participants answered each question with “yes” or “no,” providing a total GDS score of 0–15; higher scores indicated more severe depressive state. Participants with the total GDS score <6 were considered to have non-depressive state, and those with the total GDS score ≥6 were considered to have depressive state [18]. The cut-off point of 6 has been reported to have a sensitivity of 0.973 and specificity of 0.959 [18]. Depressive state was then assessed as a binary variable based on the cut-off point [18].

#### Possible confounders

As possible confounders, the following were considered: sex, age, marital status, annual family income, living alone, staying indoors, falling within a year, skin ulcer, subjective memory complaints, medical histories of diabetes, hypertension, stroke, or Parkinson’s disease, and body mass index (BMI). They were chosen based on previous studies of the associations between depressive symptoms and higher-level functional capacity [13–15]. Most confounders were provided as dichotomous variables in the questionnaire; however, age and BMI were assessed as quantitative variables. Participants were asked to provide their sex, birthdate, marital status, annual family income, and medical histories. They were also asked yes or no questions regarding living alone, staying indoors, falling within a year, skin ulcers, and subjective memory complaints. Marital status was divided into married or other. Annual family income was divided into <3 million yen or ≥3 million yen, based on the Japanese Comprehensive Survey of Living Conditions in 2012, showing that 3.079 million yen was the average for elderly people in Japan (http://www8.cao.go.jp/kourei/english/annualreport/2012/pdf/1-2-2.pdf, Accessed October 5, 2014). BMI was calculated as weight (kg) divided by the square of the height (m).

#### Statistical analyses

To remove confounding and estimate the marginal odds ratios, inverse probability of treatment weighting (IPTW) using the propensity score, which allowed some of characteristics of RCTs to be mimicked in an observational study [16], was used. Using this method, a synthetic sample was created where the distribution of measured covariates were independent of the exposure similar to that created by randomization in RCTs.

To estimate the propensity score predicting the probability of depressive state, multiple logistic regression analyses were conducted with depressive state as the outcome variable and with age, BMI, marital status, annual income, living alone, staying indoors, falling within a year, skin ulcer, subjective memory complaints, and medical histories, such as diabetes, hypertension, stroke, or Parkinson’s disease as predictors. The selection method of possible confounders [19] was used for the propensity score model of the present study.

To assess whether the propensity score models were adequately specified, their c-statistics [19] and standardized differences [16,20] were obtained. The c-statistics indicated the degree to which the propensity score model discriminated between non-depressive and depressive state. A previous study has suggested that c-statistics > 0.67 were least problematic [19]. The standardized differences indicate the degree of systematic differences in covariates between depressive state using the IPTW and propensity score. Empirically, an absolute standardized difference of <10% was used to indicate a negligible difference in the mean or prevalence of the covariates between high and non-depressive state [16].

To investigate the associations between depressive state and higher-level functional capacity, we estimated the marginal odds ratios by logistic models using generalized linear models with IPTW for propensity score and design-based standard errors [20–22]. All analyses were stratified by sex and 95% confidence intervals (CIs) were obtained. The R statistical software, version 3.1.2 [23], was used for statistical analyses.

A simple imputation using predictive mean matching in the MICE 2.13 package for the R statistical software was used to handle the missing values [24,25]. For sensitivity analyses based on missing at random assumptions, missing values were handled using multiple imputation by regression switching followed by predictive mean matching in the MICE 2.13 package to give preferred parameter estimates [24,25]. Forty copies of the data were independently analyzed [26], the resulting parameter estimates and standard errors by Rubin’s rules [27] were pooled, and odds ratios and 95% CIs were obtained.

## Results

Self-report questionnaire were distributed to 897 participants, and 616 responded (response rate 68.7%); of these, 54 were <65 years old, nine had dementia, five were receiving treatment for depression, and three did not provide their sex. Thus, 545 eligible participants were included in the final analysis. Table 1 shows the participant characteristics.

**Table 1:** Characteristics of participants.

Variables	Men (n = 233)	Women (n = 312)	Missing rate (%)
***Continuous variables***			
Age (years, mean, SD)	79.27 (6.97)	80.99 (6.19)	0
Age range (years, range)	65–97	65–94	0
Body height (cm, mean, SD)	163.49 (7.40)	150.23 (6.75)	9.17
Body weight (kg, mean, SD)	60.49 (10.32)	50.74 (9.37)	8.44
Body mass index (kg/m^2^, mean, SD)	22.62 (3.17)	22.41 (3.72)	10.28
GDS score (mean, SD)	5.67 (3.59)	5.74 (3.58)	29.36
TMIG competence score (mean, SD)	7.98 (3.67)	9.16 (3.67)	18.72
***Categorical variables***			
Married + (n, %)	190 (81.55)	113 (36.22)	0.18
Annual income in family below 300 million yen + (n, %)	78 (33.48)	132 (42.31)	21.10
Living alone + (n, %)	33 (14.16)	120 (38.46)	0.37
Staying indoor + (n, %)	36 (15.45)	35 (11.22)	4.22
Falling within a year + (n, %)	106 (45.49)	148 (47.44)	2.75
Skin ulcer + (n, %)	80 (34.33)	145 (46.47)	3.85
Subjective memory complaints + (n, %)	47 (20.17)	67 (21.47)	7.34
Diabetes + (n, %)	32 (13.73)	25 (8.01)	0
Hypertension + (n, %)	36 (15.45)	54 (17.31)	0
Stroke + (n, %)	26 (11.16)	21 (6.73)	0
Parkinson’s disease + (n, %)	10 (4.29)	7 (2.24)	0
Depressive state + (n, %)	80 (34.33)	95 (30.45)	29.36
Impaired HLFC + (n, %)	59 (25.32)	42 (13.46)	18.72

Abbreviations: GDS, geriatric depression scale; HLFC, higher-level functional capacity; SD, standard deviation; TMIG, Tokyo Metropolitan Institute of Gerontology.

A simple imputation by predictive mean matching was use to handle missing values in the following analyses. The propensity scores were estimated using propensity score models, which had c-statistics of 0.73 for men and 0.71 for women. Tables 2 and 3 summarize the standardized differences in the covariates between non-depressive and depressive state before and after the IPTW using the propensity scores. The all absolute standardized differences after the IPTW using the propensity scores were <10% and they improved from before to after. Therefore, we considered that the propensity score models were adequately specified.

**Table 2:** Means, proportions, and absolute values of standardized differences before and after IPTW with propensity scores in men.

Covariates	Depressive state (n = 116)	Non-depressive state (n = 117)	Standardized differences (%)	Depressive state	Non-depressive state	Standardized differences (%)
Age (year, mean)	78.02	80.51	36.33	79.26	79.08	2.59
Body mass index (kg/m^2^, mean)	22.54	22.67	3.92	22.47	22.44	1.13
Married + (%)	75.86	87.18	29.48	81.72	81.51	0.54
Annual income in family below 300 million yen + (%)	45.69	31.62	29.2	40.48	40.11	0.75
Living alone + (%)	18.97	9.4	27.69	13.71	14.14	1.24
Staying indoor + (%)	19.83	11.11	24.29	15.01	13.18	5.26
Falling within a year + (%)	46.55	58.97	25.07	51.82	54.04	4.45
Skin ulcer + (%)	52.59	74.36	46.41	63.19	64.57	2.87
Subjective memory complaints + (%)	23.28	20.51	6.7	22.05	18.28	9.41
Diabetes + (%)	13.79	13.68	0.32	12.26	12.14	0.37
Hypertension + (%)	14.66	16.24	4.37	14.89	15.4	1.42
Stroke + (%)	14.66	7.69	22.26	12.73	13.78	3.1
Parkinson’s disease + (%)	6.03	2.56	17.18	4.5	4.34	0.78

Abbreviations: IPTW, inverse probability of treatment weighting.

**Table 3:** Means, proportions, and absolute values of standardized differences before and after IPTW with propensity scores in women.

Covariates	Depressive state (n = 142)	Non-depressive state (n = 170)	Standardized differences (%)	Depressive state	Non-depressive state	Standardized differences (%)
Age (year, mean)	80.29	81.58	20.90	81.18	81.37	3.14
Body mass index (kg/m^2^)	22.37	22.39	0.60	22.26	22.35	2.27
Married + (%)	40.14	32.94	14.99	35.62	34.13	3.13
Annual income in family below 300 million yen + (%)	59.15	55.88	6.62	56.37	57.93	3.15
Living alone + (%)	36.62	40.59	8.16	38.76	39.68	1.88
Staying indoor + (%)	13.38	10	10.53	11.97	11.09	2.76
Falling within a year + (%)	49.3	52.35	6.1	49.78	50.77	1.98
Skin ulcer + (%)	38.03	63.53	52.75	50.99	51.7	1.42
Subjective memory complaints + (%)	32.39	15.88	39.32	23.72	24.08	0.84
Diabetes + (%)	11.97	4.71	26.49	8.51	9.23	2.53
Hypertension + (%)	14.08	20	15.79	16.43	17.91	3.93
Stroke + (%)	7.75	5.88	7.43	6.23	6.4	0.7
Parkinson’s disease + (%)	3.52	1.18	15.49	2.29	2.53	1.56

Abbreviations: IPTW, inverse probability of treatment weighting.

Table 4 shows the marginal odds ratios obtained from logistic models using IPTW for propensity score with depressive state as the exposure and impaired higher-level functional capacity as the binomial outcome. The marginal odds ratios were 2.17 (95% CI: 1.13–4.19) for men and 2.57 (95% CI: 1.26–5.26) for women. Depressive state was significantly associated with impaired higher-level functional capacity in both men and women. Similar results were obtained in the sensitivity analyses for handling missing values using multiple imputation in all models (Tables 4), which showed that our handling of missing values was reasonable.

**Table 4:** Marginal odds ratios by logistic models with depressive status as the exposure and impaired HLFC as the outcome using IPTW with propensity scores^†^ in men (n = 233) and women (n = 312).

	Odds ratios	Lower 95% CIs	Upper 95% CIs
*Men*			
Depressive state +	2.17	1.13	4.19
*Women*			
Depressive state +	2.57	1.26	5.26
*Men (sensitivity analysis using multiple imputation)*			
Depressive state +	1.99	1.02	3.90
*Women (sensitivity analysis using multiple imputation)*			
Depressive state +	2.32	1.04	5.17

Abbreviations: CI, confidence interval; HLFC, higher-level functional capacity; IPTW, inverse probability of treatment weighting.

†Propensity scores were estimated by multiple logistic regression analyses with depressive status as the outcome and the following covariates as the predictors: age, body mass index, marital status, annual income, living alone, staying indoors, falling within a year, skin ulcer, subjective memory complains, and medical histories of diabetic, hypertension, stroke, or Parkinson’s disease.

## Discussion

The present study aimed to investigate the association between depressive state and higher-level functional capacity and obtain marginal odds ratios using propensity score analyses among people requiring long-term care. The marginal odds ratios (i.e., the change in odds of impaired higher-level functional capacity if all versus no participants were exposed to depressive state) were 2.17 (95% CI: 1.13–4.19) for men and 2.57 (95% CI: 1.26–5.26) for women.

Our results support the results of previous observational longitudinal studies that have shown that depressive symptoms predicted a decline in higher-level functional capacity among the community-dwelling elderly [14,15]. In the previous studies, the cut-off point of higher-level functional capacity measured by TMIG-index of competence were 10/11 [14] and declines in scores of TMIG-index of competence at follow-up point from the base line point [15]. Specifically, the present study focused on people requiring long-term care and who have generally more risks of functional capacity than the community-dwelling elderly. In the present study, the cut-off point of higher-level functional capacity measured by TMIG-index of competence was 5/6 because of people with the total score ≤5 having a 10%–20% 1-year mortality, whereas those with the total score >5 having a <5% 1-year mortality [1]. Thus, the present study may suggest that depressive state is associated with impaired higher-level functional capacity leading to high 1-year mortality.

The previous longitidinal observational studies have shown that the depressive symptoms predicted a decline in higher-level functional capacity as a conditional effects (at an individual-level) by multiple Cox proportional hazards regression analyses [14] and by multiple logistic regression analyses [15]. The present study showed the significant associations between depressive state and impaired higher-level functional capacity and demonstrated the marginal odds ratios (at a population level) by propensity score analyses. The marginal odds ratios are also important considerations because of usefulness to apply results of the preset study to clinical settings at population level. The results of the present study is similar to the results of previous studies by RCTs demonstrating marginal effects of intensive treatment for depression on ADL [28] and IADL [29–31].

A possible biological mechanism for the associations between depressive state and impaired higher-level functional capacity may includes the occurrence of hippocampal volume loss. Previous studies have shown that depression was associated with hippocampal volume loss [32–35]. In addition, a longitudinal study demonstrated that low hippocampal volume predicted declines in IADL [36], which were a central component of higher-level functional capacity [1]. Thus, we consider that hippocampal loss may account for the associations between depressive state and impaired higer-level functional capacity. Although the present study was not designed to assess these biological mechanisms, further studies are warranted to assess whether hippocampal volume loss in depression can cause impaired higher-level functional capacity.

The present study has several limitations. First, cross-sectional design, which precludes any causal direction assessment, was used. To assess causal direction, a cohort study design is required; a previous longitudinal study has indicated that depressive symptoms predicted impaired higher-level functional capacity [14,15]. Second, despite being reported to be valid, the present study employed only subjective self-report questionnaires. Third, if some confounding covariates were missed such as genetic factors, our models would have been insufficient to create a synthetic sample with a covariate distribution that was independent of depressive state, similarly to that assigned by randomization in RCTs. However, the c-statistics and standardized differences were adequate.

The present study obtained the marginal odds ratios using propensity score in the context of an observational study; this was the strength of the present study. Propensity score analyses are easier to decide whether models can adequately adjust for covariates than regression analyses [16]. In addition, propensity score analyses are more robust to misspecification of models than regression analyses [37,38]. Second, the present study was based on a sample from outpatient care services for long-term preventive care. This sample allowed the investigation of depressive state and higher-level functional capacity in an at-risk population. Understanding the associations between depressive state and impaired higher-level functional capacity and identifying impaired higher-level functional capacity in an at-risk population can facilitate appropriate preventive intervention and sustain healthy, socially independent living.

In conclusion, the present study showed the significant associations between depressive state and impaired higher-level functional capacity among people requiring long-term care. Preventing depression may contribute to not only depressive state but also higher-level functional capacity. Finally, by helping to identify an at-risk subgroup of elderly patient, our results could lead to the possibility of identifying any decline of higher-level functional capacity; therefore, appropriate interventions could be introduced earlier, thereby preventing unnecessary sequelae.
